# An Update on the Accessibility and Quality of Online Information for Pediatric Orthopaedic Surgery Fellowships

**DOI:** 10.7759/cureus.17802

**Published:** 2021-09-07

**Authors:** Samuel A Cohen, Kevin Shea, Meghan Imrie

**Affiliations:** 1 Orthopaedic Surgery, Stanford University School of Medicine, Stanford, USA

**Keywords:** orthopaedics, quality, online, accessibility, fellowship, pediatric orthopaedics, website

## Abstract

Introduction

The internet is an important tool for applicants seeking information on pediatric orthopaedic surgery fellowship programs. Previous analysis of pediatric orthopaedic surgery fellowship websites demonstrated they were often inaccessible and incomplete. As such, the purpose of this study was to (1) perform an updated assessment of the accessibility and content of pediatric orthopaedic fellowship program websites and (2) compare the results to the previous study to discern temporal trends in website accessibility and quality.

Methods

A list of pediatric orthopaedic fellowship programs was compiled from the San Francisco Match (SF Match) and the Pediatric Orthopaedic Society of North America (POSNA) online databases. All identified websites were evaluated for (1) accessibility and (2) the presence of 12 education and 12 recruitment criteria. These criteria were determined by prior fellowship website analyses and the needs of current fellowship applicants. Website accessibility and quality were compared with previously reported metrics.

Results

Approximately 91% of pediatric orthopaedic surgery fellowship programs had a functioning website. While the SF Match and POSNA databases listed nearly identical programs, there were discrepancies in the information provided by the two databases, and individual program website links provided on both databases were often nonfunctional. Fellowship program websites contained an average of 15.1 ± 3.9 total education and recruitment criteria (range: 3 - 21). The most common education criteria featured on program websites included information about research, affiliated hospital information, and rotations. The most common recruitment criteria featured on program websites included program descriptions, contact information, and social media links. There was an increased frequency in nearly all education and recruitment criteria evaluated when compared with 2014 metrics.

Discussion

Although website accessibility and content have improved since 2014, information on pediatric orthopaedic fellowship program websites remains incomplete, with many websites failing to provide information on criteria deemed important by fellowship applicants. In addition, many discrepancies exist between the SF Match and POSNA databases, the two primary sources of information for pediatric orthopaedic fellowship applicants. Increased consistency on pediatric orthopaedic fellowship websites and both the SF Match and POSNA databases may help applicants to better assess which programs to apply to and which programs to rank highly on their match list.

## Introduction

In the past two decades, the proportion of orthopaedic surgery residents applying for fellowship training has increased dramatically. In a 2012 Fellowship and Residency Electronic Interactive Database (FREIDA) survey, 87% of orthopaedic surgery residents expressed an intention to pursue fellowship training, up from approximately 66% and 76% in 1997 and 2003, respectively [1]. The improved job prospects for fellowship-trained orthopaedic surgeons, when compared with general orthopedists, is one factor that is likely driving increased specialization within the field [1]. Pediatric orthopaedic surgery remains a competitive subspecialty desired by orthopaedic surgery residents. Pediatric orthopaedic surgery fellowship applicants currently submit an average of 18.6 applications and attend more than 13 interviews during a given application cycle [2]. As the number of applications submitted per pediatric orthopaedic surgery fellowship applicant has increased, so has the need for a centralized database where prospective applicants can learn more about the potential fellowship programs available to them.

In 2009, the Pediatric Orthopaedic Society of North America (POSNA) partnered with the San Francisco Match (SF Match) to standardize the application process for pediatric orthopaedic surgery fellowship applicants. Both the POSNA and SF Match databases currently provide a list of orthopaedic fellowship programs within the United States and Canada. In addition, both databases provide basic information about each program listed, such as the number of fellowship positions available, contact information, and website links. While background information about each program is provided on the POSNA and SF Match databases, the majority of information regarding pediatric orthopaedic fellowship programs that may be useful for prospective applicants is likely to be found on individual program websites. Both residency and fellowship applicants throughout the country have indicated that a program’s website is an important factor when deciding where to apply, where to interview, and where to rank programs during the match process [3-8]. Despite the importance of residency and fellowship program websites, they are inconsistent regarding both accessibility and content [9-12].

Davidson et al. evaluated the content and accessibility of pediatric orthopaedic surgery fellowship websites in 2013 and found that the SF Match and POSNA databases provided few direct links to fellowship websites, and individual program websites rarely conveyed the necessary information desired by applicants [13]. Nearly a decade later, the importance of an effective fellowship website is even more pressing as a result of the shift to virtual interviews due to the COVID-19 pandemic. As such, the purpose of this study was to (1) perform an updated assessment of the accessibility and content of pediatric orthopaedic fellowship program websites and (2) compare the results to the previous study in order to discern temporal trends in website accessibility and quality.

## Materials and methods

Database listings

We queried the lists of pediatric orthopaedic surgery programs on the websites of POSNA (http://www.posna.org) and SF Match (http://www.sfmatch.org) from March 13, 2021 to April 14, 2021. We extracted the number of fellowship positions available, contact information, and website links from all programs on both databases. We subsequently evaluated the congruency of information between the POSNA and SF Match databases. 

Website accessibility

A Google (http://www.google.com) search was conducted to determine the accessibility of program websites from outside the POSNA and SF Match databases. We performed two searches for each program and evaluated the first page of results (10 listings) for direct links to program websites. Two separate search terms were included in an attempt to cover the possible terms used by applicants: (1) “program name + pediatric orthopaedic fellowship” and (2) “program name + pediatric orthopaedic surgery fellowship.”

Content analysis

All accessible program websites were evaluated for information regarding fellow education and recruitment. The education and recruitment criteria being assessed were marked as present if the website provided information on the topic, regardless of the quantity or quality of the information presented. This method is consistent with website content analyses used in previous studies and was done in order to maintain objectivity [10-17]. Fellowship program websites were evaluated based on 12 criteria related to the educational experience of fellows and 12 criteria related to the recruitment of fellows. Criteria were derived from several previous fellowship website content analyses, including the Davidson et al. 2014 paper evaluating pediatric orthopaedic surgery fellowship websites, as well as fellowship websites for other medical specialties [10-16]. Examples of educational criteria include research, rotations, case descriptions, and journal club. Examples of recruitment criteria included program description, salary, application process, and current fellows. Table 1 displays all 24 criteria that were used in our content analysis. 

**Table 1:** Pediatric Orthopaedic Surgery Fellowship Education and Recruitment Variables

Education	Recruitment
Research	Description
Didactics	Contact information
Rotations	Application process
Case descriptions	SFmatch.org link
Office/clinic time	Salary
Journal club	Current fellows
Call	POSNA.org link
Meetings/conferences	Prior fellow listing
Teaching responsibilities	Prior fellow outcomes
Faculty listing	Video content
Affiliated hospital information	Location description
Quality improvement	Social media links

## Results

Website accessibility

Overall, 42 of the 46 pediatric orthopaedic fellowship programs studied had functional websites, the majority of which (23/42, 54.8%) had been updated within the past calendar year. All 42 programs with a functional website were accessible via an independent Google search when using either of the search parameters included in this study: “program name + pediatric orthopaedic fellowship” or “program name + pediatric orthopaedic surgery fellowship.”

The POSNA database lists 46 total programs while the SF Match database lists 45 programs. Forty-five programs were listed on both respective databases and a single program was listed on the POSNA database but not the SF Match database. With regards to the number of fellowship positions available, the number listed was consistent across both databases for 43/45 (95.6%) of programs listed. When comparing the 45 programs listed in both databases, contact information differed for 15 (33.3%) programs. 

Of the 46 programs listed in the POSNA database, 28 (60.9%) had website links listed on the database that properly redirected the user to the fellowship website. For the SF Match database, 14 (31.1%) programs had website links listed that properly redirected the user to the fellowship website.

Fellow education

**Figure 1:**
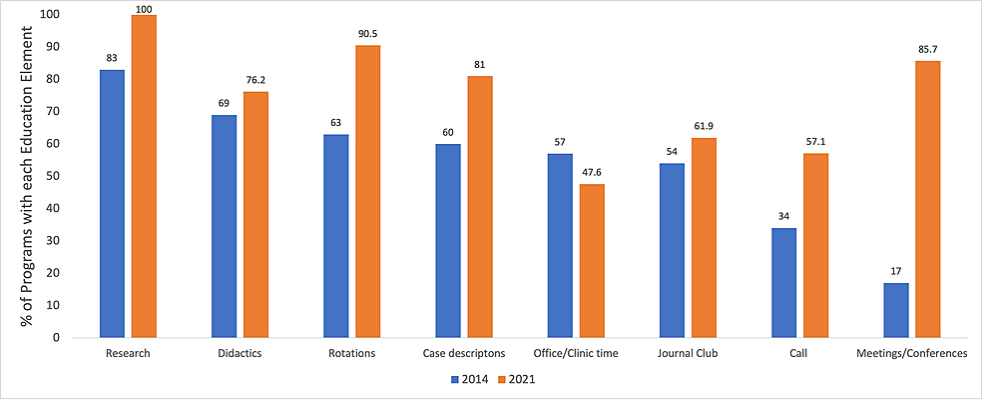
Proportion of pediatric orthopaedic fellowship program websites containing the eight educational criteria measured in both the 2014 study and the present study

Fellowship program websites contained an average of 8.8 ± 2.4 of the 12 education criteria analyzed (range: 2 - 12). The most common education criteria featured by pediatric orthopaedic fellowship websites included affiliated hospital information (100%), research (100%), and rotations (90.5%). The frequency of each of the 12 education criteria analyzed can be observed in Table 2. Figure 1 compares the frequency of the eight criteria examined in both the 2014 Davidson et al. study and the present study [13].

**Table 2:** Number (%) of Websites with Listed Information on Various Education Criteria

Education (n = 42)	n (%)
Research	42 (100)
Didactics	32 (76.2)
Rotations	38 (90.5)
Case descriptions	34 (81.0)
Office/clinic time	20 (47.6)
Journal club	26 (61.9)
Call	24 (57.1)
Meetings/conferences	36 (85.7)
Teaching responsibilities	37 (88.1)
Faculty listing	33 (78.6)
Affiliated hospital information	42 (100)
Quality improvement	5 (11.9)

Fellow recruitment

**Figure 2:**
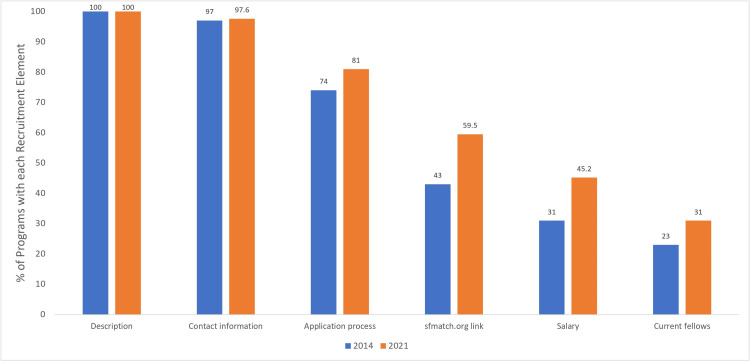
Proportion of pediatric orthopaedic fellowship program websites containing the six recruitment criteria measured in both the 2014 study and the present study

Fellowship program websites contained an average of 6.3 ± 2.1 of the 12 recruitment criteria analyzed (range: 1 - 10). The most common recruitment criteria featured by pediatric orthopaedic fellowship websites included program description (100%), contact information (97.6%), and social media links (85.7%). The frequency of each of the 12 recruitment criteria analyzed can be observed in Table 3. Figure 2 compares the frequency of the six criteria examined in both the 2014 Davidson et al. study and the present study [13].

**Table 3:** Number (%) of Websites with Listed Information on Various Recruitment Criteria

Recruitment (n = 42)	n (%)
Description	42 (100)
Contact information	41 (97.6)
Application process	34 (81.0)
SFmatch.org link	25 (59.5)
Salary	19 (45.2)
Current fellows	13 (31.0)
POSNA.org link	6 (14.3)
Prior fellow listing	18 (42.9)
Prior fellow outcomes	14 (33.3)
Video content	17 (40.5)
Location description	16 (38.1)
Social media links	36 (85.7)

Total recruitment and education content score

**Figure 3:**
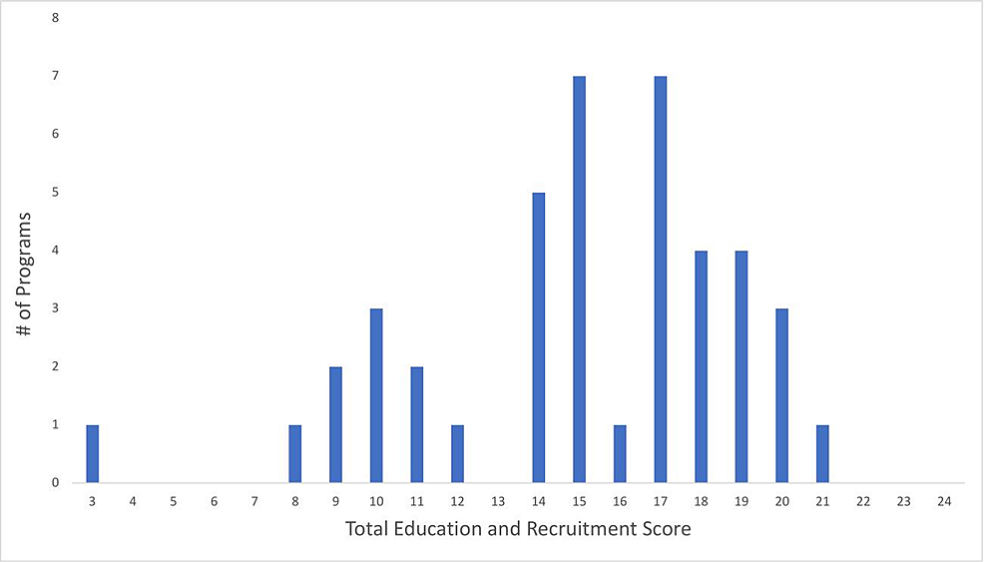
Distribution of total education and recruitment content scores for all pediatric orthopaedic fellowship program websites analyzed

Fellowship program websites contained an average of 15.1 ± 3.9 total education and recruitment criteria (range: 3 - 21). The distribution of total education and recruitment content scores can be observed in Figure 3. 

## Discussion

The purpose of this study was to evaluate the accessibility and quality of current pediatric orthopaedic surgery fellowship websites. With regards to accessibility, all programs with functional websites were accessible via an independent Google search, a dramatic improvement since 2013 [13]. However, the two primary databases on which information about pediatric orthopaedic surgery fellowships are listed (POSNA and SF Match) remain incomplete and inconsistent. With regards to website quality, our results indicate that websites contained an average of 15.1 out of a possible 24 education and recruitment criteria (range: 3 - 21), which suggests inconsistency in fellowship websites, as well as ample room for improvement. Increased consistency on pediatric orthopaedic fellowship websites may help applicants to better assess which programs to apply to and which programs to rank highly on their match list. 

Many prior studies have demonstrated that both residency and fellowship applicants consider program websites to be an important factor when deciding which programs to apply to, interview at, and ultimately, rank highly [4, 6, 17-18]. However, in order for a fellowship website to serve as an effective recruitment tool, it must be accessible. Despite increased accessibility overall via an independent Google search compared to 2013, the two most common databases that applicants use to learn more information about prospective fellowship programs, POSNA and SF Match, often fail to provide working links to a program’s affiliated fellowship program website. In addition, there is often conflicting contact information when comparing the POSNA and SF Match databases that may confuse applicants seeking program-specific information. Working with program directors to ensure that contact information and website links uploaded to both the POSNA and SF Match databases are accurate and up-to-date may help applicants more easily access and navigate the webpages of fellowship programs of interest to them. 

With regards to educational content analyzed on fellowship program websites, our results indicate an increase in frequency for seven of the eight criteria that were measured in both 2013 and 2021 [13]. The large increase in the proportion of websites containing information about meetings/conferences (+68.7% compared to 2013), rotations (+27.5%), and case descriptions (+21%) suggest that fellowship programs have actively improved their websites in the past eight years; however, there is still work left to do, with less than half of programs providing information about office/clinic time and only slightly more than half of programs providing information about call, both of which have been cited as important factors that are often missing from program websites by previous graduate medical education applicants [6]. An example of a program that conveyed education information to prospective fellows is the Children’s Hospital of Philadelphia (CHOP) (http://www.chop.edu/pediatric-fellowships/orthopedic-surgery-clinical-fellowship) fellowship website. This website contains a section titled “A typical week for a clinical orthopaedic fellow at CHOP,” which provides prospective fellows with information about their day-to-day schedule, including didactic sessions, operating room time, clinic, and call. This type of day-to-day (and often hour-by-hour) breakdown was not present on the majority of pediatric orthopaedic fellowship program websites, but it was highly informative and may benefit prospective applicants who are attempting to envision themselves as future fellows.

With regards to recruitment content analyzed on fellowship program websites, there were improvements in the proportion of websites containing information about the application process (+7% compared to 2013), salary (+14.2%), and current fellows (+8%) [13]. However, less than half of websites provided information about previous fellows (42.9%), previous fellow outcomes (33.3%), and location descriptions (38.1%). Providing applicants with information about job outcomes post-fellowship, as well as a description of the location of the program, could be beneficial to programs and applicants alike, given that both factors have been cited as important to residency applicants when deciding where to apply [6, 18]. An example of a program that provided recruitment information to prospective fellows is the Children’s Hospital Colorado (http://medschool.cuanschutz.edu/orthopedics/education/fellowships/pediatric-orthopedic-surgery-fellowship) fellowship website. One feature of the Children’s Hospital Colorado website is an alumni database that provides the name, year, and current position of previous fellows from 1981-2020. This type of alumni database provides applicants with insight as to the types and locations of post-graduate outcomes that may be important in their decision-making process. Another program website with many of the recruitment criteria we studied is the University of California, San Francisco (UCSF) website (http://orthosurgery.ucsf.edu/education/fellowships/UCSF-pediatric-orthopaedic-surgery-fellowship.html). The UCSF website contains a nearly seven-minute welcome video that introduces prospective applicants to many facets of the program through the lens of many of the current faculty. The video also highlights diversity and inclusion, as well as community outreach efforts, that may be of interest to potential applicants. 

Improving the accessibility and content of pediatric orthopaedic fellowship websites may benefit both prospective applicants and fellowship programs by ensuring that applicants make the most informed decisions about where to apply. In addition, the criteria analyzed in this study would not take extraordinary resources to implement on program websites, with the majority of the criteria remaining constant over time and only a few criteria, such as current fellows, prior fellow listing, and prior fellow outcomes, requiring updates on a yearly basis. As such, after an initial investment of resources to improve the fellowship program website, there would be minimal future action required to maintain website quality.

There are limitations to our study. First, the list of education and recruitment criteria analyzed in this study is not validated. There may be other criteria important to fellowship applicants that we did not assess; however, the criteria used in this study are derived from other studies that evaluated fellowship website content and accessibility and were deemed to be relevant to the current fellowship application process by the study team [10-12, 15]. Another limitation is that we evaluated program websites for the presence of education and recruitment criteria but did not assess the quality of the criteria, except to provide a few examples of education and recruitment content. However, the binary scoring system we implemented has been used in all previous fellowship website content analyses that the criteria in our study were based on [10-16]. Finally, websites may constantly be updated by fellowship programs. As such, changes in website content since the content analyses took place would not be captured in this cross-sectional study.

## Conclusions

In conclusion, since 2014, there have been improvements with regards to both website accessibility and education and recruitment content. However, the website content remains highly variable, and there is further improvement required for websites to reach their full information potential. Efforts to improve pediatric orthopaedic surgery fellowship program websites by incorporating various education and recruitment criteria analyzed in this study would not require tremendous investment but may prove beneficial to both applicants and programs.
